# Comparative performance of ^11^C-methionine and ^11^C-choline PET/CT in primary hyperparathyroidism: Impact of adenoma weight on PET detectability

**DOI:** 10.1007/s00259-026-08116-6

**Published:** 2026-08-14

**Authors:** Laura García Belaustegui, Magdalena de la Higuera López-Frías, Jorge de Abajo-Larriba, Andrés Basanta Mota, Juan Fernando Bastidas, María Isabel Morales Lozano, Fabiana Grissanti, Javier Arbizu

**Affiliations:** 1 Department of Nuclear Medicine, Clínica Universidad de Navarra, Madrid, Spain; 2 Department of Endocrinology, Clínica Universidad de Navarra, Madrid, Spain; 3 Department of Otorhinolaryngology, Clínica Universidad de Navarra, Madrid, Spain

**Keywords:** Primary hyperparathyroidism, PET/CT, [^11^C]methionine, [^11^C]choline, Parathyroid adenoma

## Abstract

**Purpose:**

To evaluate the diagnostic performance of [¹¹C]methionine (MET) PET/CT for preoperative localisation in primary hyperparathyroidism (PHPT) with negative or inconclusive first-line imaging, and to compare it with [¹¹C]choline (CHO) PET/CT in a head-to-head analysis, focusing on determinants of adenoma detectability.

**Methods:**

Fifty-one patients with biochemically confirmed PHPT underwent MET PET/CT following inconclusive conventional imaging. In a subgroup of 25 patients, CHO PET/CT was also performed. Imaging findings were correlated with surgical and histopathological results. Detection rates and positive predictive values (PPV) were calculated, and semiquantitative parameters, including standardized uptake value (SUV) and lesion-to-background ratio (LBR), were analysed. Associations between PET findings, biochemical markers, and adenoma weight were explored.

**Results:**

MET PET/CT achieved a patient-based detection rate of 76.5% with a PPV of 88.2%. In the head-to-head subgroup, CHO detected more lesions than MET (27 vs. 14; *p* = 0.0025) and identified all histologically confirmed adenomas, with comparable PPVs (90.0% vs. 77.8%). CHO demonstrated significantly higher SUV and LBR values. Adenoma weight was significantly associated with PET findings: MET-positive adenomas were significantly heavier than MET-negative adenomas, whereas CHO maintained high lesion detection even in adenomas weighing 0.5 g or less. Additionally, a trend towards higher uptake with increasing PTH and calcium levels was observed for CHO SUVmax.

**Conclusion:**

Adenoma weight appears to be a major determinant of PET detectability in PHPT. The ability of CHO PET/CT to detect adenomas weighing 0.5 g or less, which may remain occult on MET, provides a plausible explanation for its superior lesion detection, while both tracers maintain a high positive predictive value. These findings further support the use of choline-based PET imaging in patients with inconclusive first-line studies.

## Introduction

Primary hyperparathyroidism (PHPT) is a common endocrine disorder and the leading cause of hypercalcaemia [1], most often resulting from a solitary parathyroid adenoma, although hyperplasia, multiple adenomas, and, more rarely, carcinoma may also be implicated [2]. The diagnosis is established biochemically by elevated or inappropriately normal parathyroid hormone (PTH) levels in the presence of hypercalcaemia or high-normal serum calcium concentrations [2].

Parathyroidectomy remains the only curative treatment for patients meeting surgical criteria, and the widespread adoption of minimally invasive approaches has increased the need for accurate preoperative localisation. Conventional first-line imaging includes cervical ultrasonography (US), [^99m^Tc]Tc-MIBI scintigraphy with single photon emission computed tomography (SPECT/CT), and computed tomography (CT) [3]. However, these techniques show variable diagnostic performance and may be limited in cases of multiglandular disease, ectopic lesions, or concomitant thyroid pathology [4–8].

In this context, positron emission tomography (PET) has emerged as a valuable second-line modality when conventional imaging results are inconclusive. Initially, [^11^C]methionine (MET), a radiotracer that exploits increased amino acid transport and protein synthesis in hyperfunctioning parathyroid tissue [9], demonstrated encouraging results [10]. More recently, choline-based PET tracers—[^18^F]fluorocholine (FCH) and [^11^C]choline (CHO)—which reflect increased phospholipid metabolism in hyperfunctioning glands [11], have gained attention owing to their favourable imaging characteristics [12]. The clinical implementation of these tracers varies according to local radiopharmaceutical availability and production infrastructure, as the short physical half-life of CHO requires on-site cyclotron production.

The present study aimed to evaluate the diagnostic performance of MET PET/CT for preoperative localisation of hyperfunctioning parathyroid glands in patients with PHPT and negative or inconclusive first-line imaging, and to compare its lesion detection rate with that of CHO PET/CT in a head-to-head subgroup. In addition, we explored associations between imaging findings, clinical parameters, and gland characteristics to better understand the factors underlying differences in detectability.

## Materials and methods

### Patient population

This retrospective study included 51 patients with biochemically confirmed PHPT—defined as hypercalcaemia in the presence of elevated or abnormal PTH levels who met surgical criteria and underwent MET PET/CT at our centre between February 2010 and April 2020 after negative or inconclusive cervical US and/or [^99m^Tc]Tc-MIBI SPECT/CT. Patients with secondary or tertiary hyperparathyroidism or familial hypocalciuric hypercalcaemia were excluded. In a subset of 25 patients, CHO PET/CT was additionally performed as part of this research (code CUN-RAD-2020-01). The study was approved by the regional ethics committee of the Government of Navarre, classified by the Spanish Agency for Medicines and Health Products, and all patients provided written informed consent.

### PET/CT procedure

All examinations were performed using standardized acquisition protocols on a hybrid PET/CT system (Siemens Biograph mCT 64 scanner). Patients fasted for at least 6 h prior to imaging and received an intravenous injection of either MET (370–550 MBq) or CHO (200–650 MBq), in accordance with previously published protocols [13, 14].

Image acquisition was performed approximately 20 min after MET administration and 5 min after CHO injection. Low-dose, non-contrast CT was used for attenuation correction and anatomical localisation, covering the neck and upper thorax.

### Image interpretation and quantification

PET/CT images were independently reviewed by two experienced nuclear medicine physicians, blinded to surgical and histopathological data. Focal tracer uptake suggestive of hyperfunctioning parathyroid tissue was classified according to anatomical location as typical (adjacent to the thyroid gland) or ectopic (intrathyroidal, paraesophageal, lower cervical or mediastinal). Discrepancies were resolved by consensus.

In addition, a semiquantitative analysis was performed using manually defined three-dimensional volumes of interest (VOIs). For each suspected lesion, a VOI was positioned over the lesion to obtain SUVmax and SUVmean. A separate 1-cm³ VOI was placed in the contralateral thyroid lobe, whenever feasible, to obtain thyroid SUVmean. The lesion-to-background ratio (LBR) was defined as lesion SUVmax divided by thyroid SUVmean [9]. In visually negative MET scans, a VOI was positioned at the anatomical site where the lesion was identified on CHO PET/CT in order to assess MET uptake at that location and calculate LBR.

### Surgery and histopathological confirmation

Surgical treatment, predominantly minimally invasive parathyroidectomy (MIP), was guided by PET/CT findings, with the surgical approach adapted when multiple suspected lesions, ectopic glands or concomitant thyroid pathology were present on imaging.

Surgical success was defined as excision of the PET/CT-localised lesion with histopathological confirmation of parathyroid adenoma or hyperplasia and a > 50% intraoperative decline in PTH levels, in accordance with the Miami criteria [15]. Lesions with discordant localisation between PET/CT and surgery or those histologically confirmed as normal parathyroid tissue were considered false positives.

Although biochemical cure was not used as the reference standard for lesion-based analysis, all patients underwent postoperative biochemical follow-up. Biochemical cure was defined as sustained normocalcaemia with appropriate PTH levels for at least six months after surgery [16].

### Statistical analysis

Continuous variables were expressed as mean ± standard deviation or median (interquartile range) and categorical variables as counts and percentages. Group comparisons were performed using parametric or non-parametric tests as appropriate.

Paired lesion-based comparisons between MET and CHO were performed using McNemar’s test, while differences in semiquantitative PET parameters (SUVmax, SUVmean and LBR) were analysed using paired t-tests or Wilcoxon signed-rank tests. Statistical significance was defined as a two-sided *p* < 0.05. All analyses were performed using Stata (version 15).

Associations between PET positivity and preoperative biochemical markers were explored for MET only, as all CHO scans were positive.

## Results

### Sociodemographic and clinical characteristics

The study included 51 patients with confirmed PHPT who met surgical criteria, with a median age of 60 years and a female predominance (Table 1). Concomitant thyroid pathology and osteoporosis were frequent, and renal involvement—mainly nephrolithiasis—was observed in a substantial proportion of patients, whereas vertebral fractures were less common.

**Table 1:** Sociodemographic, clinical and biochemical characteristics of the study population (*n* = 51)

Demographic and clinical characteristics	
Age, years	60 (14–81)
Female sex	38 (74.5%)
Previous parathyroid surgery	4 (7.7%)
Concomitant thyroid pathology	23 (44.2%)
Osteoporosis	27 (51.9%)
Nephrolithiasis	21 (40.4%)
Vertebral fractures	4 (7.7%)
Biochemical parameters	
PTH, pg/mL	157 (68–416)
Serum calcium, mg/dL	11.0 ± 0.9
Serum phosphate, mg/dL	2.64 ± 0.63
25-hydroxyvitamin D, ng/mL	31.8 (11–69)
Serum creatinine, mg/dL	0.8 (0.4–2.1)
Urinary calcium, 24 h, mg	279 (25–559)

Data are presented as median (range) or mean ± standard deviation (SD), as appropriate. Serum calcium values are albumin-corrected. The number of available cases was 48 for PTH, serum calcium and serum creatinine; 38 for serum phosphate; 31 for vitamin D; and 24 for 24-h urinary calcium. Reference ranges were 8.6–10.2 mg/dL for serum calcium and 15–65 pg/mL for PTH

Renal function was generally preserved, while vitamin D levels and 24-hour urinary calcium excretion showed marked interindividual variability (Table 1).

### Surgical outcomes

A total of 56 parathyroid nodules were identified, most of which were located adjacent to the right lower thyroid pole (Table 2). Seven ectopic lesions were observed, including mediastinal, lower cervical and paraesophageal locations. Forty-five nodules (corresponding to 43 patients) underwent surgical resection, predominantly by MIP, with an adapted surgical approach required in a minority of cases. All resected nodules were positive on at least one PET/CT examination. Surgical data were unavailable for 8 patients: seven were lost to follow-up, while one patient did not undergo surgery due to an incidental lung cancer detected on PET/CT and died during follow-up.

**Table 2:** Surgical and histopathological outcomes

Patients and nodules	
Patients	51
Parathyroid nodules identified	56
Ectopic nodules - Mediastinal - Lower cervical - Paraesophageal	7 (12.5%) 3 3 1
Nodules surgically treated	45
Nodules not resected or without adequate follow-up	11
Surgical approach	
MIP	39
Adapted surgical approach	6
Histopathological findings (resected nodules)	
- Adenoma - Hyperplasia - False positives findings	38 3 4
Adenoma weight, g	0.59 (0.07–2.63)
Intraoperative PTH, pg/mL	25.8 (4–197)
Postoperative outcomes (6-month follow-up)	
Patients achieving biochemical cure	42 (100%)
Postoperative PTH, pg/mL	59 (19–144.5)
Postoperative serum calcium, mg/dL	9.44 ± 0.43

Data are presented as median (range) or mean ± standard deviation (SD), as appropriate. Available data: adenoma weight (*n* = 36), intraoperative PTH (*n* = 42), postoperative PTH (*n* = 41), and postoperative serum calcium (*n* = 42)

Histopathological analysis confirmed parathyroid adenoma in most resected lesions, with a smaller number of hyperplastic glands and histologically normal parathyroid tissue (Table 2). Adenoma weight showed moderate variability. Intraoperative PTH levels declined appropriately after resection. At 6-month follow-up, all patients with available data achieved normocalcemia. Although postoperative PTH levels remained elevated in a subset of patients due to secondary causes, the median value remained within the reference range.

### PET/CT results

#### Diagnostic performance of MET PET/CT

MET PET/CT demonstrated 42 foci of uptake suggestive of hyperfunctioning parathyroid tissue in 39 of 51 patients, corresponding to a patient-based detection rate of 76.5% (Table 3). Surgical and histopathological verification was available for 34 PET-positive nodules, of which 30 were confirmed as pathological parathyroid tissue at the anatomical site indicated by PET, yielding a PPV of 88.2%.

**Table 3:** Diagnostic performance of MET PET/CT (*n* = 51)

PET-positive patients	39 (76.5%)
Total PET-positive nodules	42
PET-positive nodules with surgical verification	34
Histologically confirmed pathological parathyroid tissue at PET-indicated site	30
False-positive findings, n - Histologically normal parathyroid tissue (no adenoma identified) - Mislocalisation (adenoma identified at a different site)	4 2 2
PPV (surgically verified cohort)	88.2%

Detection rate was calculated on a patient basis; PPV calculated among nodules with histopathological verification

Four PET-positive foci were classified as false positives. Two occurred in a single patient with multinodular goitre, in whom the adenoma was ultimately identified at a different location at surgery. Two additional cases involved the resection of histologically normal parathyroid tissue at PET-suspected sites, with no adenoma found. Notably, both patients achieved biochemical cure after follow-up.

The assessment of sensitivity was limited due to the lack of systematic surgical verification in PET-negative patients and incomplete follow-up in certain cases.

#### Head-to-head comparison of MET and CHO PET/CT

Among the 25 patients who underwent both MET and CHO PET/CT, imaging was performed within 6 months in most cases. In six patients, CHO PET/CT was performed later during clinical follow-up after an initial negative MET PET/CT, resulting in an interval of more than 6 months between examinations. Nevertheless, no significant differences were observed in preoperative PTH (mean difference 5.3 pg/mL; 95% CI − 19.1 to 29.7; *p* = 0.60) or serum calcium levels (mean difference 0.25 mg/dL; 95% CI − 0.14 to 0.64; *p* = 0.16) between the two examinations in these six patients. These findings support biochemical stability and the comparability of both imaging studies despite the temporal interval.

CHO detected a greater number of nodules than MET (27 vs. 14), and all nodules identified on MET were anatomically consistent with the CHO findings (Table 4). Of the 27 CHO-positive nodules, 20 (from 19 patients) underwent surgical resection, yielding 18 histologically confirmed adenomas and two histologically normal parathyroid glands. The latter corresponded to those cases previously described, in which positivity on both tracers was identified and a biochemical cure was achieved during follow-up.

**Table 4:** Head-to-head comparison of MET and CHO PET/CT (*n* = 25)

	MET	CHO
Nodules detected	14	27
Histologically confirmed adenomas (true positives)	7	18
False-positive findings	2	2
Detection rate (adenoma-based)	38.9% (7/18)	100% (18/18)
PPV	77.8% (7/9)	90.0% (18/20)

Detection rates and PPV were calculated in the surgically verified subset of 20 CHO-positive nodules (18 adenomas and 2 histologically normal glands)

MET correctly identified 7 of the 18 adenomas, corresponding to a lesion-based detection rate of 38.9% and a PPV of 77.8%. In contrast, CHO PET/CT detected all 18 adenomas, resulting in a detection rate of 100% and a PPV of 90.0%. This represented a 2.57-fold higher lesion detection rate for CHO compared with MET (95% CI 1.44–4.59; *p* = 0.0025).

**Fig. 1:**
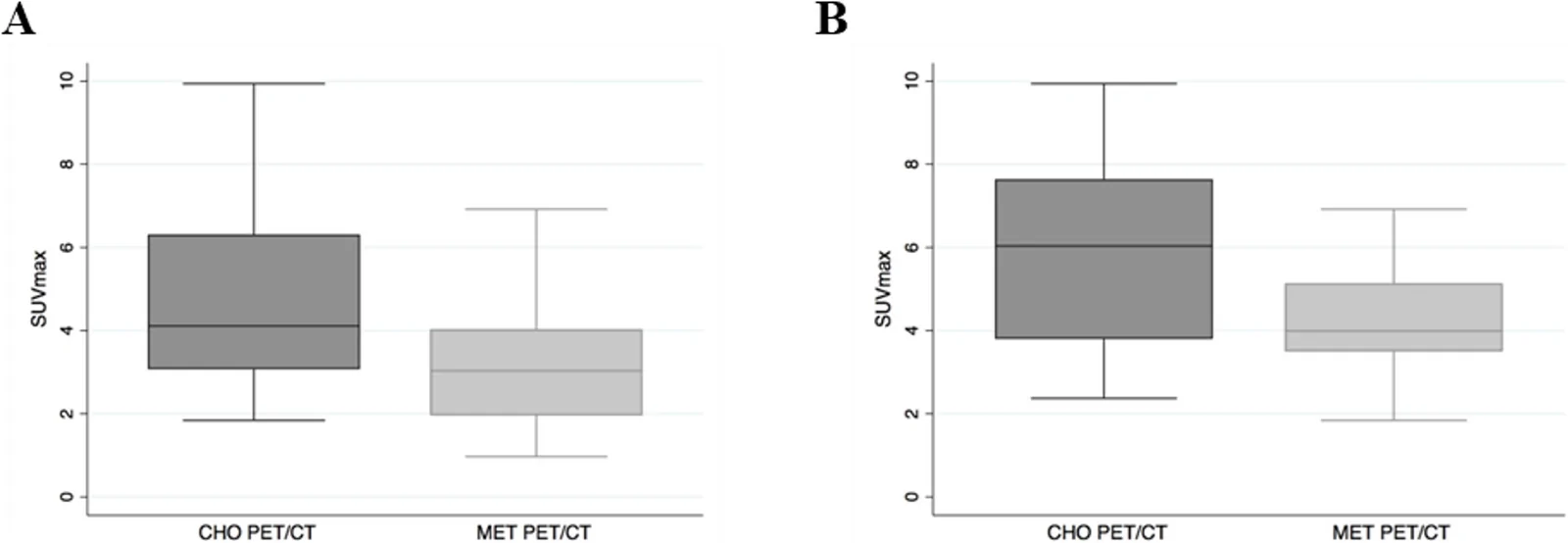
Comparison of SUVmax values between CHO and MET PET/CT. (**A**) All 27 nodules included in the head-to-head analysis. For visually negative MET scans, SUVmax was measured at the anatomical location corresponding to the lesion identified on CHO PET/CT. (**B**) Analysis restricted to the 14 nodules detected by both tracers

Semiquantitative analysis demonstrated significantly higher tracer uptake on CHO compared with MET (Fig. 1). Across all analysed nodules (*n* = 27), SUVmax and SUVmean were higher on CHO than on MET (SUVmax: 4.76 vs. 3.21; mean difference + 1.55; 95% CI 1.02–2.07; *p* < 0.0001; SUVmean: 3.27 vs. 2.32; mean difference + 0.95; 95% CI 0.57–1.33; *p* < 0.0001). Similar results were observed in the subgroup of nodules detected by both tracers (*n* = 14), with higher uptake on CHO (SUVmax: 5.97 vs. 4.28; mean difference + 1.69; 95% CI 0.75–2.62; *p* = 0.0018; SUVmean: 3.84 vs. 2.88; mean difference + 0.96; 95% CI 0.31–1.61; *p* = 0.0072).

**Fig. 2:**
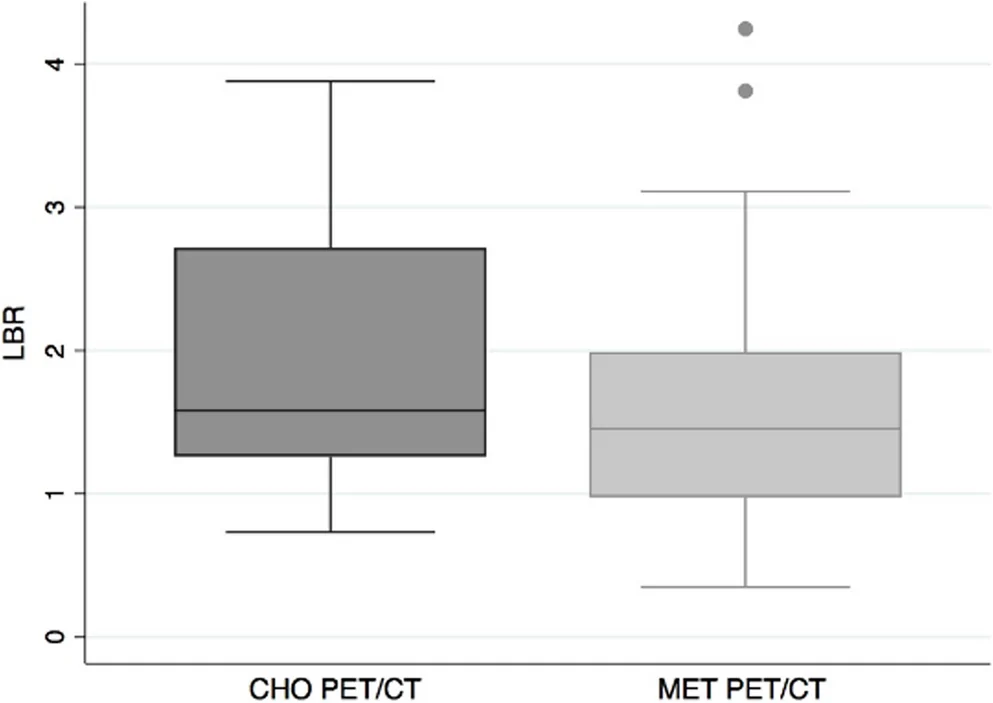
Comparison of lesion-to-background ratios (LBR) between CHO and MET PET/CT. LBR was calculated as lesion SUVmax divided by thyroid SUVmean. Results are shown for all 27 nodules included in the head-to-head analysis and for the subgroup of nodules detected by both tracers

Beyond absolute uptake values, LBR analysis further supported these findings (Fig. 2). CHO demonstrated significantly higher LBRs than MET in the overall cohort (median 1.58 vs. 1.14; *p* = 0.0003), a finding confirmed by paired analysis. This difference persisted in the subgroup of nodules detected by both tracers, with higher LBRs on CHO (median 2.71 vs. 1.71; *p* = 0.0076). Taken together, these results indicate superior lesion-to-background contrast for CHO compared with MET.

#### PET/CT findings in relation to biochemical markers and adenoma weight

The association between PET/CT positivity and preoperative biochemical markers was evaluated in all surgically treated patients (*n* = 43), stratified as MET-positive or MET-negative. All MET-negative patients underwent surgery exclusively after localisation by CHO PET/CT. A similar analysis could not be performed for CHO, as all patients in our cohort had positive findings.

Mean total serum calcium concentrations were comparable between groups (10.95 vs. 11.13 mg/dL; *p* = 0.51), whereas a trend towards higher preoperative PTH levels was observed in MET-positive compared with MET-negative patients (180.1 vs. 151.6 pg/mL; *p* = 0.088).

**Fig. 3:**
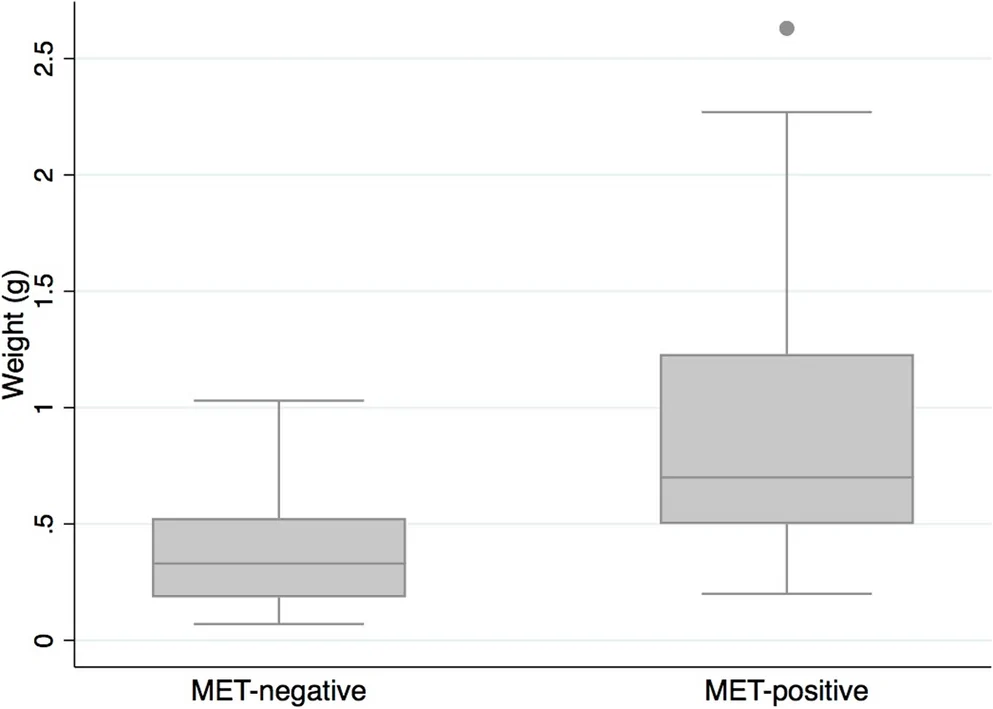
Distribution of adenoma weight according to MET PET/CT findings. Boxplots show adenoma weight in MET-positive and MET-negative lesions. MET-positive adenomas were significantly heavier than MET-negative adenomas (median 0.70 g vs. 0.36 g; *p* = 0.0042)

**Fig. 4:**
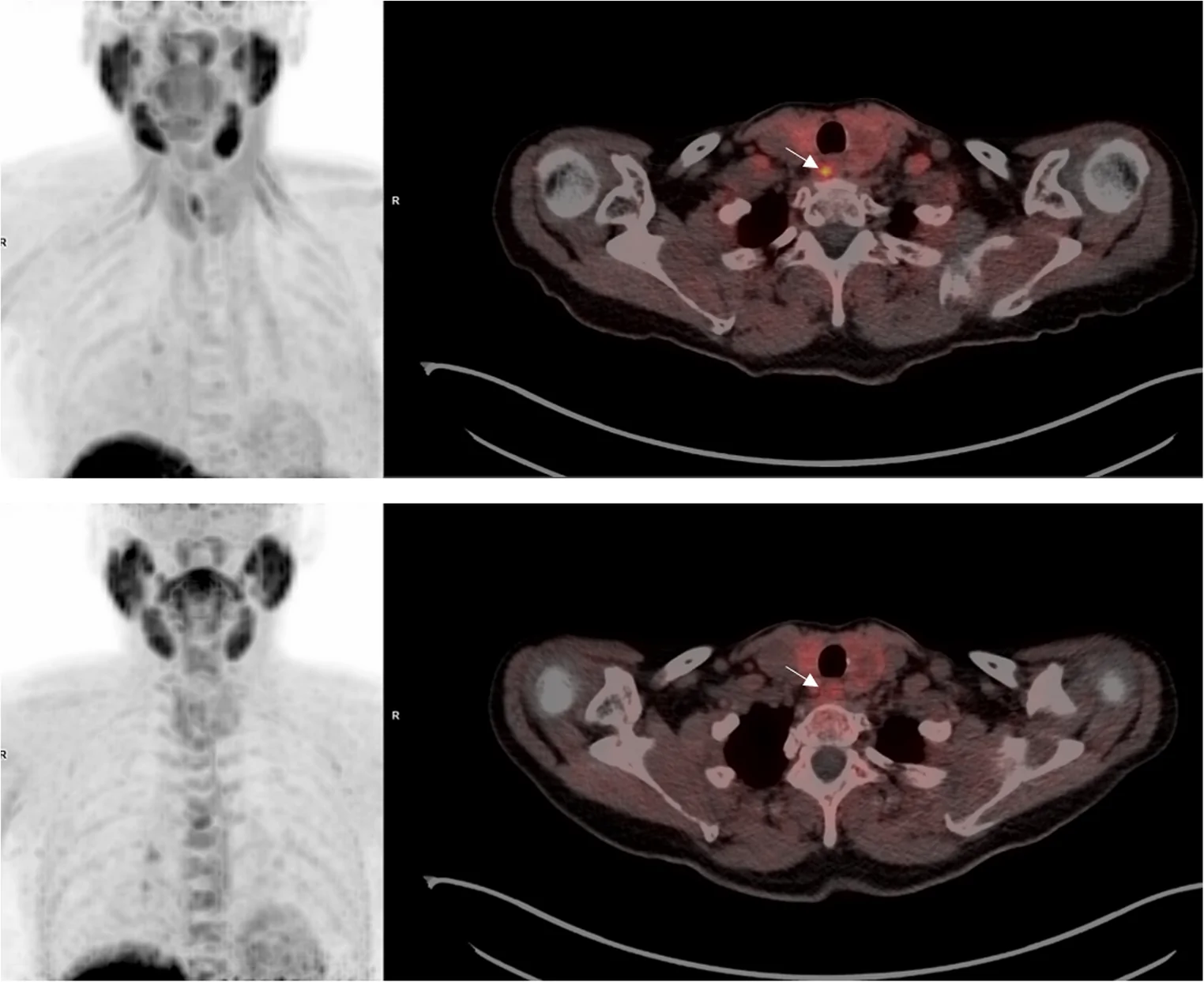
Histologically confirmed parathyroid adenoma located posterior to the right thyroid lobe. Upper row: CHO PET/CT demonstrates focal tracer uptake corresponding to the adenoma (SUVmax = 4.3; LBR = 1.9). Lower row: the lesion is not visually identified on the corresponding MET PET/CT, showing only background-level uptake (SUVmax = 2.7; LBR = 1.0). Surgical pathology confirmed an adenoma weighing 0.54 g

In contrast, adenoma weight differed according to MET status (Fig. 3). MET-positive adenomas were heavier than MET-negative ones (median 0.70 g vs. 0.36 g; *p* = 0.0042). Mean adenoma weight was nearly twice as high in the MET-positive group (0.95 g vs. 0.44 g), indicating a predominance of larger adenomas among MET-detected nodules. Notably, 10 of the 11 MET-negative adenomas weighed ≤ 0.5 g and were resected only after localisation by CHO PET/CT. A representative example of a lower-weight adenoma detected by CHO PET/CT but not by MET PET/CT is shown in Fig. 4.

**Fig. 5:**
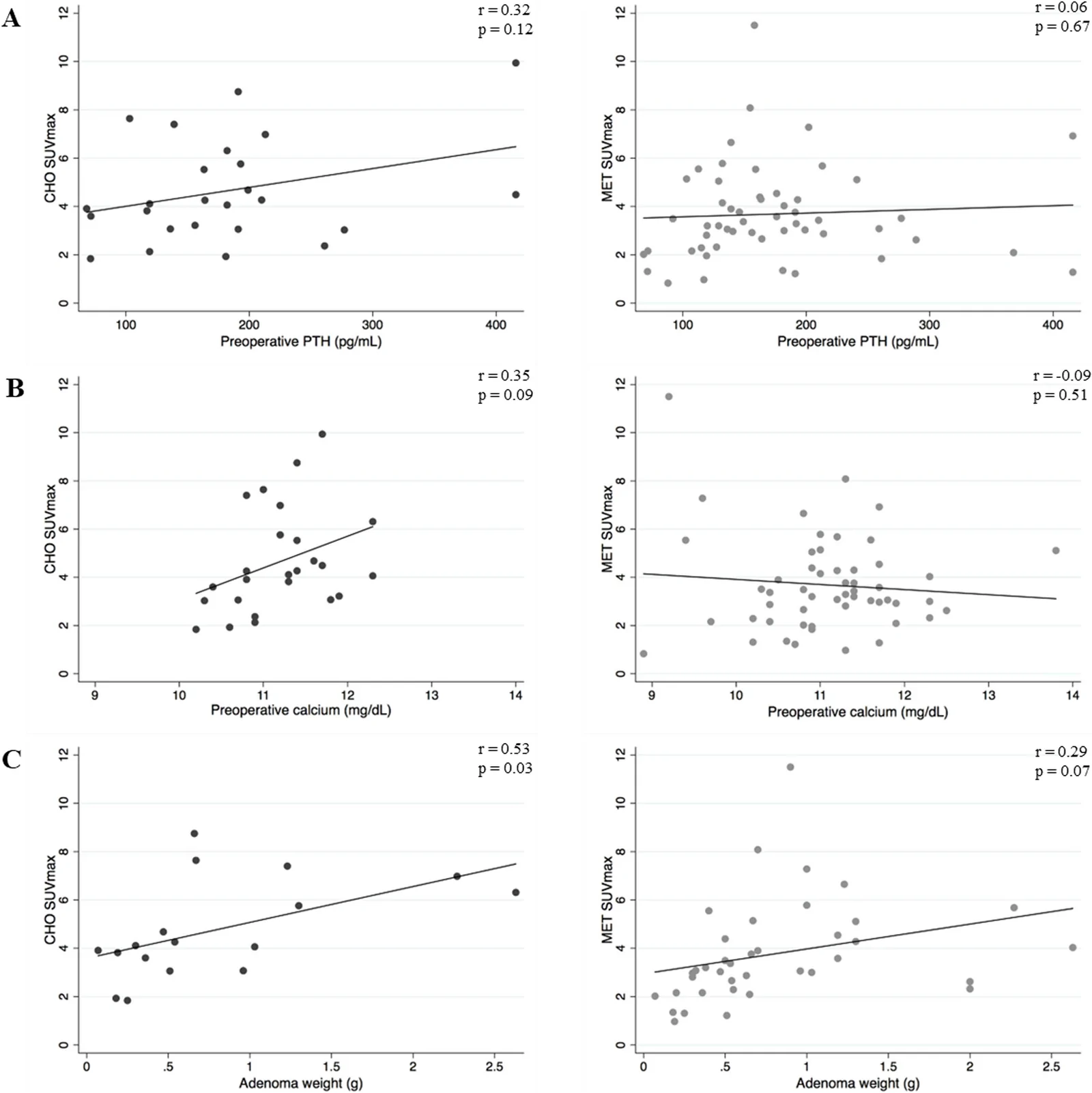
Scatter plots showing the relationship between SUVmax and preoperative PTH (**A**), serum calcium (**B**), and adenoma weight (**C**) for CHO and MET PET/CT. Correlation coefficients (r) and p-values are shown for each tracer

Beyond qualitative PET positivity, correlation analyses were performed between semiquantitative PET parameters (SUVmax and SUVmean) and preoperative biochemical markers, as well as adenoma weight, for both MET and CHO (Fig. 5). No statistically significant correlations were observed between tracer uptake and serum calcium or PTH levels. However, CHO SUVmax showed a non-significant trend towards higher uptake with increasing PTH and calcium levels.

In contrast, adenoma weight showed a stronger association with tracer uptake, particularly for CHO. CHO SUVmax showed a moderate and statistically significant correlation with gland weight (*r* = 0.53, *p* = 0.03). For MET, weaker associations with adenoma weight were observed (SUVmax: *r* = 0.29, *p* = 0.07).

## Discussion

The present study corroborates the elevated detection rate of MET PET/CT in biochemically confirmed PHPT patients with negative or equivocal first-line imaging and demonstrates the superiority of CHO over MET in a head-to-head comparison. It provides additional insight concerning the enhanced lesion-to-background contrast and the stronger relationship between PET/CT findings and adenoma weight when using CHO.

Accurate preoperative localisation is essential to facilitate MIP in patients with PHPT. In this study, CHO demonstrated higher detection rates compared with MET in patients with negative or inconclusive first-line imaging, particularly for lower-weight adenomas. These findings position CHO PET/CT as a highly effective imaging modality in challenging cases. Although FCH has become the most widely adopted choline tracer owing to its longer physical half-life and broader availability, our findings demonstrate that CHO PET/CT remains a highly effective imaging modality in challenging cases and an established alternative in institutions with on-site cyclotron facilities.

In the head-to-head comparison, CHO detected all surgically confirmed adenomas and demonstrated a 2.57-fold higher lesion detection rate than MET (95% CI 1.44–4.59; *p* = 0.0025). Despite this difference, both tracers showed similarly high PPVs, indicating that the main advantage of CHO lies in improved lesion detection. These results are consistent with the only available direct comparison between MET and CHO PET/CT [12] and with a recent meta-analysis comparing FCH with MET PET/CT [17], both of which also demonstrated superior diagnostic performance of choline-based tracers with comparable PPVs.

Semiquantitative analyses also favoured CHO in our cohort, with higher SUV values and LBRs than MET. Because both tracers were labelled with the same radionuclide, these differences are unlikely to reflect technical factors and more likely represent tracer-specific biological behaviour [17]. Our findings align with previous reports using choline-based tracers. In the study by Mathey et al. [9] using FCH instead of CHO, choline PET demonstrated higher uptake and improved lesion-to-background contrast compared with MET, together with superior sensitivity for parathyroid localisation. In our series, CHO demonstrated significantly greater tracer uptake than MET, with a mean SUVmax difference of + 1.55 (95% CI 1.02–2.07; *p* < 0.0001) and a mean SUVmean difference of + 0.95 (95% CI 0.57–1.33; *p* < 0.0001). These findings are comparable to those reported by Mathey et al. for FCH versus MET.

A key finding of this study is that adenoma weight emerged as a major determinant of PET/CT detectability. MET-positive adenomas had a median weight almost twice that of MET-negative lesions (0.70 g vs. 0.36 g; *p* = 0.0042). The difference was driven by the capacity of CHO to identify smaller glands, including the majority of adenomas weighing ≤ 0.5 g that were not visible on MET PET/CT. Semiquantitative analysis corroborated these observations, showing a moderate and statistically significant correlation between CHO SUVmax and adenoma weight, and weaker but directionally similar associations for MET. To our knowledge, few studies have explored the relationship between PET findings and adenoma weight. Building on previous observations from choline-based series [18], our study is the first to evaluate this issue in the context of a direct intraindividual comparison between methionine and choline PET/CT. Our results support the concept that adenoma weight plays an important role in determining PET detectability and may contribute to the observed differences in lesion detection between both tracers.

In contrast, neither qualitative PET findings nor semiquantitative parameters were significantly associated with preoperative biochemical variables in our cohort, although non-significant trends were observed, including higher PTH levels in MET-positive patients and increasing CHO SUVmax values with rising PTH and calcium levels. This lack of significant associations differs from some prior reports [19] and may reflect the limited sample size.

Overall, both tracers showed a high PPV, with only four false-positive findings observed in our cohort. Two of these occurred in a patient with multinodular goitre who underwent only MET PET/CT, where the presence of multiple thyroid nodules may have acted as a confounding factor, an aspect that lies beyond the scope of the present study. The remaining two lesions, positive on both MET and CHO, were reported histologically as normal parathyroid tissue; however, postoperative follow-up demonstrated normalisation of serum calcium and PTH levels, indicating biochemical cure.

In our study, lesion-based evaluation relied on intraoperative findings and histopathology as reference standards, in line with common practice in PHPT imaging studies [20, 21]. Nevertheless, clinical success is ultimately defined by biochemical cure—namely sustained normocalcaemia for at least six months after surgery with appropriate normalisation of PTH levels—in accordance with current guidelines [16, 22]. Discrepancies between imaging findings and histopathology may therefore reflect sampling limitations, incomplete excision of hyperfunctioning tissue, or the presence of enlarged but histologically normal parathyroid glands with increased functional activity, whose resection may result in clinical improvement; as previously described by Krawitz et al. [23]. In light of these considerations, we chose to classify lesions with histologically normal parathyroid tissue as false positives for the lesion-based analysis, while considering postoperative biochemical outcomes when interpreting PET/CT findings at the patient level.

This study has several limitations. First, its retrospective, single-centre design and relatively small sample size, particularly in the head-to-head subgroup, may have limited the precision of some estimates and may have reduced statistical power for exploratory correlation analyses. Nevertheless, the observed differences in lesion detection between CHO and MET were substantial and statistically significant. Second, the comparative analysis is subject to potential selection bias, as not all patients who underwent MET PET/CT also underwent CHO PET/CT. However, most patients in the head-to-head subgroup were prospectively and consecutively enrolled, and the paired design using two tracers labelled with the same radionuclide minimised technical and interindividual variability. Third, in six patients, CHO PET/CT was performed more than six months after MET PET/CT. While interval-related changes in gland morphology or tracer behaviour cannot be entirely excluded, stable PTH and serum calcium levels between examinations support the comparability of the paired imaging studies. Finally, as in most PET/CT studies in PHPT, histopathological confirmation was primarily available for imaging-positive lesions, precluding reliable estimation of overall sensitivity. We therefore focused on lesion detection rates and positive predictive values, which are less susceptible to this form of verification bias.

## Conclusions

Adenoma weight emerged as a major determinant of PET detectability and tracer uptake in primary hyperparathyroidism, whereas biochemical disease severity was not significantly associated with either parameter in our cohort. The ability of CHO PET/CT to detect adenomas weighing less than 0.5 g, which may remain occult on MET PET/CT, provides a plausible explanation for its superior lesion detection. These findings further support the use of choline-based PET imaging in patients with inconclusive first-line imaging. Further prospective studies are warranted to validate these results and to better define which patients may benefit from earlier use of choline PET/CT.

## Data Availability

The datasets generated and/or analysed during the current study are available from the corresponding author on reasonable request. The study was approved by the regional ethics committee of the Government of Navarre and classified by the Spanish Agency for Medicines and Health Products (AEMPS). This study was performed in accordance with the principles of the Declaration of Helsinki. Written informed consent was obtained from all participants included in the study. Not applicable. The authors declare no competing interests.
